# Supplementary material for: Predicting visual function by interpreting a neuronal wiring diagram
Source: Nature. 2024 Oct 2;634(8032):113–23. doi: 10.1038/s41586-024-07953-5 (PMC11446822; doi:10.1038/s41586-024-07953-5)
Supplement: Supplementary file 6 — CRF and ERF predictions for individual TmY4 and TmY9 cells. Analogous to Supplementary Data 3, but for TmY target types. Shown are the top four monosynaptic pathways, the strongest pathway passing through each of the top ten intermediary types (ranking from Extended Data Fig. 7), and the trisynaptic pathway Tm1–TmY–Dm3–TmY (see the section entitled Prediction of spatial normalization). [file 41586_2024_7953_MOESM6_ESM.zip › DataS4/TmY9q/720575940624599304.pdf]

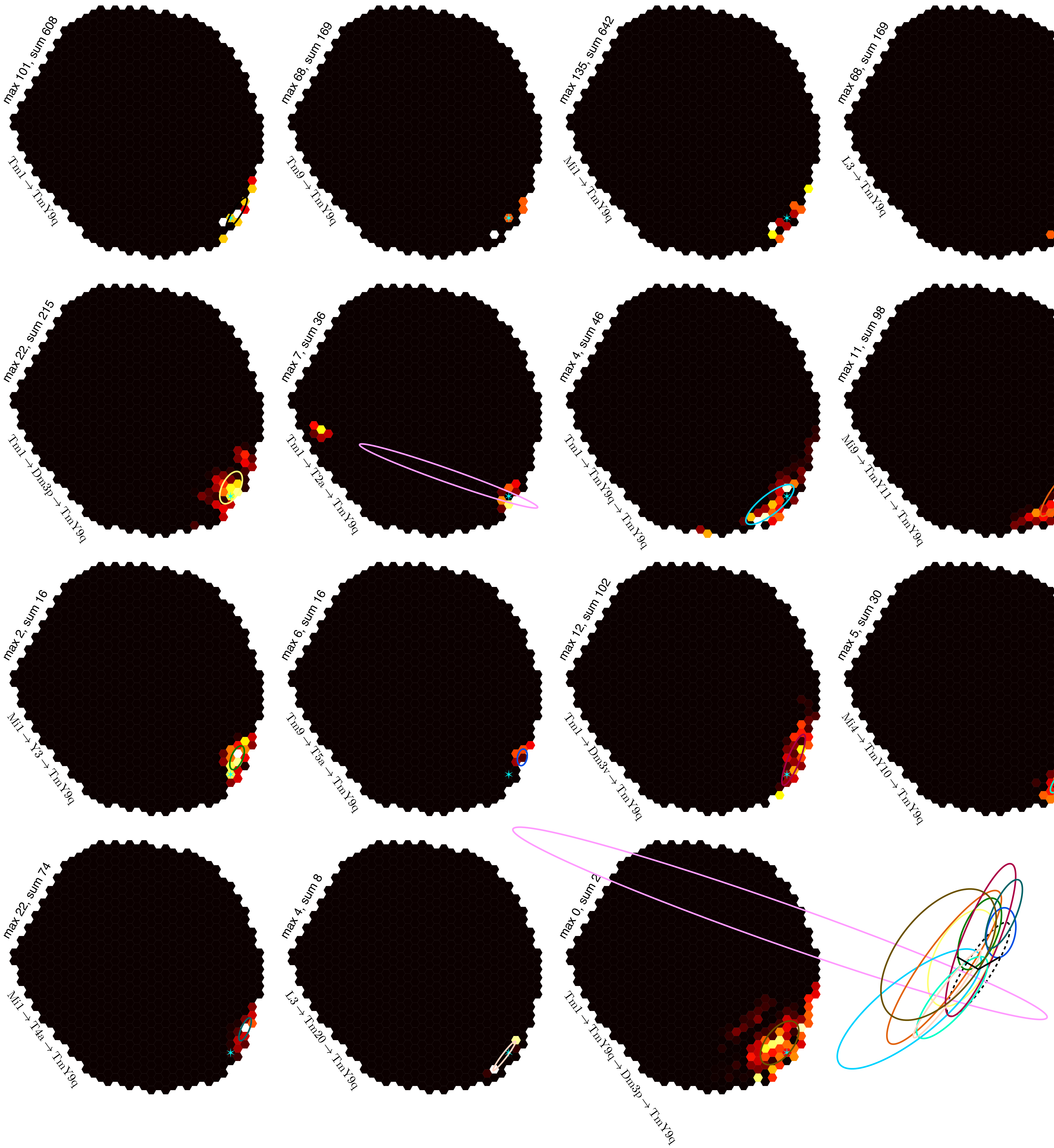

max 101, sum 608
Tm1 → TmY9q
max 68, sum 169
Tm9 → TmY9q
max 135, sum 642
Mi1 → TmY9q
max 68, sum 169
L3 → TmY9q
max 22, sum 215
Tm1 → Dm3p → TmY9q
max 7, sum 36
Tm1 → T2a → TmY9q
max 4, sum 46
Tm1 → TmY9q → TmY9q
max 11, sum 98
Mi9 → TmY11 → TmY9q
max 2, sum 16
Mi1 → Y3 → TmY9q
max 6, sum 16
Tm9 → T5a → TmY9q
max 12, sum 102
Tm1 → Dm3v → TmY9q
max 5, sum 30
Mi4 → TmY10 → TmY9q
max 22, sum 74
Mi1 → T4a → TmY9q
max 4, sum 8
L3 → Tm20 → TmY9q
max 0, sum 2
Tm1 → TmY9q → Dm3p → TmY9q
